# An Overview of the Genetics and Genomics of the *Urochloa* Species Most Commonly Used in Pastures

**DOI:** 10.3389/fpls.2021.770461

**Published:** 2021-12-13

**Authors:** Rebecca Caroline Ulbricht Ferreira, Aline da Costa Lima Moraes, Lucimara Chiari, Rosangela Maria Simeão, Bianca Baccili Zanotto Vigna, Anete Pereira de Souza

**Affiliations:** ^1^Center for Molecular Biology and Genetic Engineering (CBMEG), University of Campinas (UNICAMP), Campinas, Brazil; ^2^Embrapa Gado de Corte, Brazilian Agricultural Research Corporation, Campo Grande, Brazil; ^3^Embrapa Pecuária Sudeste, Brazilian Agricultural Research Corporation, São Carlos, Brazil; ^4^Department of Plant Biology, Biology Institute, University of Campinas (UNICAMP), Campinas, Brazil

**Keywords:** *Brachiaria*, genetic studies, genomic tools, molecular breeding, tropical forage grasses

## Abstract

Pastures based on perennial monocotyledonous plants are the principal source of nutrition for ruminant livestock in tropical and subtropical areas across the globe. The *Urochloa* genus comprises important species used in pastures, and these mainly include *Urochloa brizantha*, *Urochloa decumbens*, *Urochloa humidicola*, and *Urochloa ruziziensis*. Despite their economic relevance, there is an absence of genomic-level information for these species, and this lack is mainly due to genomic complexity, including polyploidy, high heterozygosity, and genomes with a high repeat content, which hinders advances in molecular approaches to genetic improvement. Next-generation sequencing techniques have enabled the recent release of reference genomes, genetic linkage maps, and transcriptome sequences, and this information helps improve our understanding of the genetic architecture and molecular mechanisms involved in relevant traits, such as the apomictic reproductive mode. However, more concerted research efforts are still needed to characterize germplasm resources and identify molecular markers and genes associated with target traits. In addition, the implementation of genomic selection and gene editing is needed to reduce the breeding time and expenditure. In this review, we highlight the importance and characteristics of the four main species of *Urochloa* used in pastures and discuss the current findings from genetic and genomic studies and research gaps that should be addressed in future research.

## Introduction

**Figure 1:**
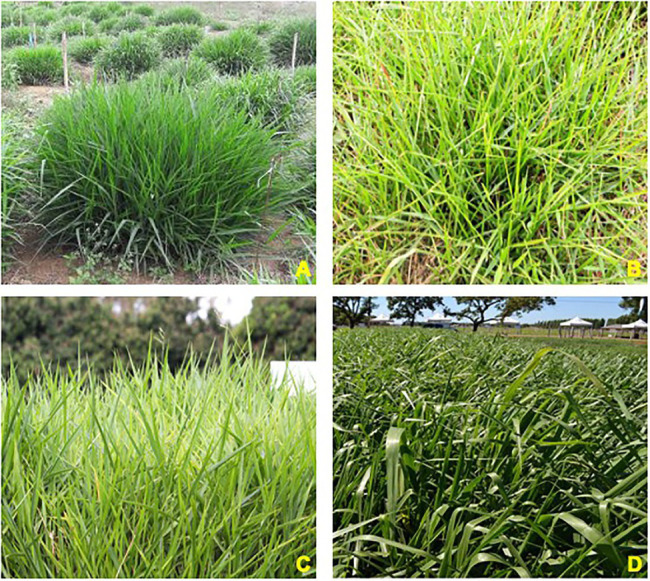
Main *Urochloa* species most commonly used in tropical pastures. **(A)** *Urochloa ruziziensis*, **(B)** *U. humidicola* cv. Tupi, **(C)** *U. decumbens* cv. Basilisk, and **(D)** *U. brizantha* cv. Piatã. Source of the photographs: Embrapa.

*Urochloa* P. Beauv. comprises important species used as forage in subtropical and tropical regions (Renvoize et al., 1996) and is mainly represented by *Urochloa brizantha* (Hochst. ex A. Rich.) R.D. Webster (syn. *Brachiaria brizantha* Hochst. ex A. Rich), *Urochloa decumbens* (Stapf) R.D. Webster (syn. *Brachiaria decumbens* Stapf), *Urochloa ruziziensis* (R. Germ. & Evrard) Crins (syn. *Brachiaria ruziziensis* R. Germ & Evrard), and *Urochloa humidicola* (Rendle) Morrone & Zuloaga (syn. *Brachiaria humidicola* Rendle; Torres González and Morton, 2005). These four species are the species considered in this review (Figure 1).

These species are native to East and Central Africa and were introduced to tropical America in the 1960s. Due to their rusticity, adaptability to areas of low fertility, tolerance to cattle trampling, good carrying capacity, and nutritional value, these species have become the main tropical forage grasses cultivated in the world and currently occupy a prominent position in the economic sector related to the seed and beef/milk markets (Jank et al., 2014). In addition, these grasses provide a reduction in production costs and are the most economical alternative in ruminant feeding in Brazil and worldwide (Ribeiro et al., 2016).

Currently, climate change and human population growth are increasing the demand for the global production of foods (Montagu, 2020), including milk and meat. Given this scenario, investment in genomic research to develop advanced breeding resources and tools is necessary to support the rapid and continuous development of improved tropical forage grasses to be used in pastures to feed livestock (Pereira et al., 2018; Simeão et al., 2021).

Compared with other important grasses, such as wheat and rice, the genetic and genomic knowledge of *Urochloa* species used in pastures remains restricted. For instance, the application of marker-assisted selection (MAS) is still limited in breeding programs, and only two sequenced genomes of diploid genotypes of *U. ruziziensis* are currently available (accessed on June 17, 2021^1^; Pessoa-Filho et al., 2019; Worthington et al., 2021). Some intrinsic characteristics, including the reproductive mode and polyploidy, as well as limited budgets and the relatively recent breeding programs directly contribute to this scenario (Pereira et al., 2018). In addition, each species has its own characteristics and specific needs, and thus, these different species require the application of different strategies and methods, as well as specialized research teams.

Despite the above-mentioned findings, the advancement of genomic technologies, such as large-scale genotyping, efficient computational resources, and appropriate statistical methods, has allowed a considerable increase in the genomic knowledge of *Urochloa* species as well as other polyploid crops in recent years. Fortunately, this knowledge is expected to be further deepened by combining multiomics strategies, including phenomics and transcriptomics, with machine learning and artificial intelligence approaches (Haas et al., 2017; Varshney et al., 2021). Consequently, there is great potential for more productive cultivars with high nutritional quality, resistance to pests, and diseases and tolerance to abiotic stresses to be launched more quickly, efficiently, and sustainably in the near future.

In this review, we summarize the importance and main characteristics of tropical forage grasses of the *Urochloa* genus used in pastures, as well the advancement of genomic resources, including molecular markers, transcriptome sequences, genome references, genomic regions of interest, and the status of molecular breeding. Our goal is to share the main information with the scientific community and breeders to increase the knowledge of the available genetic resources and expose the existing gaps that need to be filled to achieve greater genetic gains and increase the efficiency of molecular breeding.

## Economic Importance of Tropical Forage Grasses

Pastures have a great impact on the world’s economy. The livestock sector and associated market chains employ 1.3 billion people worldwide and contribute to the livelihoods of 600 million smallholder farmers (Thornton, 2010). Two (milk and beef) of the five agricultural commodities with the highest global economic value are directly related to cattle livestock, which evidences the high aggregated value of these important global assets (Rao et al., 2015). According to the Food and Agriculture Organization of the United Nations (FAO), the gross production value (GPV) of cattle meat and milk from whole fresh cows was estimated to have exceeded $493 billion in 2018.^2^

Forage grasslands, which are used to feed livestock, are estimated to represent 68% of the total agricultural area, which corresponds to one quarter of the global land area (Gordon, 2018, see footnote 2). Such crops are economically significant to all world regions, but tropical forage grasses are mainly grown in Africa and South America (Maass et al., 2015). In 2018, the land area under permanent meadows and pastures in Africa was estimated to have exceeded 842 Mha, and livestock contributes mainly to the livelihoods of small farmers and poor rural families (Adnew et al., 2021). In total, African cattle herds are estimated to have more than 361 million heads with an annual production of more than 6 million tons of meat (2018, see footnote 2).

The South American grasslands include Brazil, southern Paraguay, northeastern Argentina, and all of Uruguay, and the land area under permanent meadows and pasture is estimated to be more than 439 Mha (see footnote 2). Brazil has more than 149 Mha of areas that are destined for pastures, and the Brazilian cattle herd, which comprises more than 214 million heads, is one of the largest commercial cattle herds in the world and represents almost 60% of the South American cattle herds. In 2019, South America produced 16 million tons of beef and exported 96 thousand tons, with an exportation value of $338 million (see footnote 2).

Tropical forage seeds also impact the South American economy because Brazil is the largest producer, consumer and exporter of this product. In this scenario, the seed market of tropical forage grasses in Brazil is a business that amounts to more than $200 million a year. The legal trade deals with 120 thousand tons of pure and viable seeds each year, and more than 74% of these seeds belong to *Urochloa* spp. Of this total, more than 20% of the seeds are exported, and their main destinations are Latin America (particularly Mexico, Colombia, and Venezuela), Africa, and Asia (Jank et al., 2014).

The competitiveness of the South American livestock system in both the domestic and international markets consists of meat production being based on a pasture system. Compared with a confined cattle system, this system requires less investment and has a lower maintenance cost, and the system also provides comfort and animal welfare and ensures better meat quality and increased food security. The international scenario consisting of an increased demand for quality meat and the record number of health problems in countries that traditionally export this product have allowed Brazil to consolidate itself in the international market (Jank et al., 2011).

The human global population currently equals approximately 7.8 billion and is likely to increase to 9–10 billion in the next 30 years, which would consequently require an increase in the production of meat, milk, and other livestock products (de Oliveira Silva et al., 2016; Simeão et al., 2021). However, the conversion of land to support livestock occurs at the expense of land that is currently used by crops, forests, and native vegetation. Additionally, the arable land available for food production will decrease to 0.15 ha per person, and climate changes during the next 50 years pose a threat to the productive efficacy and welfare of livestock across the globe (Britt et al., 2018). This scenario imposes a number of challenges on livestock management worldwide: increasing productivity, changing soil and pasture management, and focusing on improving the productive potential of the areas. However, despite the great effort exerted by world research institutions toward the release of new forage grass cultivars, the number of commercial cultivars available and effectively used in pastures remains low (Jank et al., 2011; Table 1), particularly compared with that of other plants of economic importance. For example, for coffee (*Coffea arabica*), which is also a polyploid perennial species, 137 cultivars are registered with the Brazilian Ministry of Agriculture, Livestock and Supply, whereas only 30 cultivars of *Urochloa* pasture species are available (MAPA, 2021).

**Table 1:** Commercially available *Urochloa* cultivars in the world.

Cultivar	Species	Release year	Institution
Kennedy	*U. ruziziensis*	1966	*QHPLC*
Marandu	*U. brizantha*	1984	Embrapa
La Libertad (MG-4)	*U. brizantha*	1987	ICA
Basilisk	*U. decumbens*	1996	CSIR (now CSIRO)
Mulato I	Hybrid of *U. ruziziensis* and *U. brizantha*	2000	CIAT
Xaraés	*U. brizantha*	2003	Embrapa
Mulato II	Hybrid of *U. ruziziensis*, *U. brizantha* and *U. decumbens*	2005	CIAT
BRS Piatã	*U. brizantha*	2006	Embrapa
BRS Tupi	*U. humidicola*	2009	Embrapa
BRS Paiaguás	*U. brizantha*	2013	Embrapa
Mixe Drwn 12	Hybrid of *U. ruziziensis* and *U. brizantha*	2013	Bramixe, S.A. de C.v
Mixe LN 45	Hybrid of *U. ruziziensis* and *U. brizantha*	2013	Bramixe, S.A. de C.v
Braúna MG 13	*U. brizantha*	2014	Comércio e Indústria Matsuda Importadora e Exportadora LTDA
BRS RB331 Ipyporã	Hybrid of *U. ruziziensis* and *U. brizantha*	2017	Embrapa
BRS Ybaté	*U. brizantha*	2020	Embrapa
BRS Integra	*U. ruziziensis*	2020	Embrapa
Cayana	Hybrid of *U. ruziziensis* and *U. brizantha*	2020	Barenbrug do Brasil Sementes LTDA
Convert 330	Hybrid of *U. ruziziensis* and *U. brizantha*	2021	Barenbrug do Brasil Sementes LTDA
BARG156 780 J	Hybrid of *U. ruziziensis* and *U. brizantha*	2021	Barenbrug do Brasil Sementes LTDA

Source: Embrapa and MAPA. *CIAT, International Center for Tropical Agriculture; CSIR, Council for Scientific and Industrial Research, now CSIRO; CSIRO, Commonwealth Scientific and Industrial Research Organization; Embrapa, Brazilian Agricultural Research Corporation; ICA, Institute of Agricultural Sciences at the Federal University of Minas Gerais; MAPA, Brazilian Ministry of Agriculture, Livestock and Supply; QHPLC, Queensland Herbage Plant Liaison Committee*.

The current *Urochloa* spp. breeding programs focus on the development of new technologies capable of positively impacting livestock in tropical regions, which have lower inputs of resources (water, energy, fertilizers, and pesticides). The related efforts should include the development of traditional and modern breeding techniques, including genomic and phenomic tools, to achieve the goals in a timely manner.

## Summary of Taxonomy and Biology

*Urochloa* is a genus with approximately 135 species (Stevens, 2001) that belong to the family Poaceae, subfamily Panicoideae, Paniceae tribe, and subtribe Melinidinae (Salariato et al., 2010). Among the species that comprise this genus, some stand out for their agronomic and economic value and are used as pastures in tropical countries: *Urochloa brizantha*, *U. decumbens*, *U. ruziziensis*, and *U. humidicola*. Of these, *U. brizantha*, which grows in approximately 50 million hectares in Brazil, is the most commonly used forage grass in tropical pastures (Jank et al., 2014).

The taxonomic aspects of *Urochloa* forage grasses are full of complications and ambiguities due the morphological characteristics adopted as a diagnosis of the genus. Initially, Trinius (1826) considered these species to belong to a section of the *Panicum* genus, but at that time, the species were described as belonging to the genus *Brachiaria* due to its racemose primary branches (Grisebach, 1853). Later, Renvoize et al. (1996) classified the *Brachiaria* genus into nine groups based on the inflorescence and panicle morphology, and the most common commercial species were assigned to two taxonomic groups: Group 5, which consisted of *B. brizantha*, *B. ruziziensis*, and *B. decumbens*, and Group 6, which consisted of *B. humidicola* and *B. dictyoneura*.

The most important morphological characteristics considered to separate *Brachiaria* from other genera were the adaxial orientation of the spikelet and the racemose primary branches (Torres González and Morton, 2005). However, the weakness of the characteristics used to separate *Brachiaria* from *Urochloa* was discussed (Webster, 1987, 1988; Morrone and Zuloaga, 1992, 1993; Ashalatha and Nair, 1993; Veldkamp, 1996), and the *Brachiaria* genus was restricted to species with disarticulation at the base of the upper anthecium and having a smooth and shiny muticous upper anthecium. Consequently, most species of the *Brachiaria* genus were reclassified as belonging to the *Urochloa* genus (e.g., Webster, 1988; Morrone and Zuloaga, 1992, 1993; Veldkamp, 1996; Veldkamp, 2004; Torres González and Morton, 2005). In this review, we refer to these species as belonging to the *Urochloa* genus.

*Urochloa* forage grasses are perennial monocotyledonous plants originally from Africa that are propagated both vegetatively and from seeds (Hopkinson et al., 1996). Due to genetic plasticity, these plants are able to adapt to various soil and climate conditions and thus grow in a wide variety of habitats, ranging from swamps to light forest shades and semideserts, but mostly in savannas. These species have a high capacity for regrowth and high persistence in conditions of intense or frequent grazing. This capacity is directly related to the fact that these plants develop under strong pressure from the trampling of large animals, which are common in African savannas. In addition, tropical forage grasses show high rates of growth and biomass production due to the C4 photosynthetic pathway, which involves several biochemical and anatomical adjustments to accumulate additional CO_2_ compared with C3 photosynthesis (Batistoti et al., 2012).

Each commercial species has different strengths and weaknesses that need to be addressed through plant breeding. For example, *U. decumbens* was the first large-scale *Urochloa* cultivar planted in Brazil due to its excellent adaptation to acidic and nutrient-poor soils but is highly susceptible to spittlebugs attack such as *Notozulia entreriana* (Berg, 1879) (Valério and Nakano, 1987, 1988, 1989; Holmann and Peck, 2002). Currently, *U. brizantha* is the most representative of the cultivated pasture species in Brazil due to its greater resistance to spittlebugs (Jank et al., 2014), but its main disadvantage is its low tolerance to poorly drained soils. *U. humidicola* is resistant to poorly drained soils but has low nutritional value and low seed production and is susceptible to rust infection. Moreover, *U. ruziziensis* has the best nutritional value; however, this species is susceptible to spittlebug and does not adapt to acidic soils or tolerate long dry periods (Rao et al., 1996). More details of each species are shown in Table 2.

**Table 2:** General characteristics of the main species of *Urochloa* used in pastures.

	*U. brizantha*	*U. decumbens*	*U. humidicola*	*U. ruziziensis*
Common name	Bread grass/Palisade grass	Signal grass	Koronivia grass	Congo grass/Ruzi grass
Natural Distribution	Tropical Africa	Central and East Africa	East and South-East Africa	East of the Democratic Republic of the Congo; Rwanda and Burundi
Chromosome number and ploidy level	2n = 4x = 36	2n = 4x = 36	2n = 6x, 7x or 9x = 36 to 54	2n = 2x = 18
Genome Size (Gpb)	1.4	1.6	1.9	0.6
Predominant reproductive system	Facultative apomixis	Facultative apomixis	Facultative apomixis	Sexual
General morphology	Rachis narrow, crescentic; spikelets borne in a single row; glumes and lower lemma with cartilaginous texture; erect, tufted growth habit and longer leaf blades; inflorescences with 2 to 12 racemes; height in the range of 1.5 to 2.5 m.	Rachis ribbon-like; upper lemma nipped at the tip; spikelets borne in two rows; glumes and lower lemma with membranous texture; decumbent growth habitat and with lanceolate leaf blades; height in the range of 0.6 to 1 m.	Rachis very narrow, almost triquetrous; upper lemma nipped at the tip; stoloniferous growth habit; wiry culms, and three racemes in the inflorescence; height up to 1 m.	Rachis broadly winged; upper lemma striate; spikelets borne in two rows; glumes and lower lemma with a membranous texture; decumbent habitat with lanceolate leaf blades; inflorescences with 3 to 6 racemes.
Positive attributes	High productivity; tolerance to spittlebugs; drought resistance; good quality forage; ability to grow in shade.	Good performance under shade; high productivity under intense use; tolerance to aluminum; low fertility; good forage quality.	Adaptation to low-fertility and flood soils; some spittlebug resistance; strongly stoloniferous habit with ability to root at stolon nodes.	Fast growth early in the wet season; high seed production potential; good quality forage; ease of establishment.
Negative attributes	Need for moderately fertile soils; low adaptation to poorly drained soils; susceptibility to foliar blight.	Susceptibility to spittlebug and foliar blight; toxin production (sporidesmin); low adaptation to poorly drained soils.	Low seed production at low altitudes; low dry matter digestibility; susceptibility to rust infection.	Low competitiveness with weeds; susceptible to spittlebug and foliar blight; need for well-drained fertile soils.

The data are based on the information provided by Rao et al. (1996), Renvoize et al. (1996) and Simeão et al. (2021).

In addition, the above-mentioned species present some biological challenges that need to be better understood, and these include variable chromosomal behavior, different modes of inheritance (disomic, polysomic or a mixture of both as in segmental allopolyploids), highly heterozygous and high-repeat-content genomes, and variable levels of ploidy and reproductive modes (apomictic and sexual) within the same species. Breeding programs depend on this knowledge to perform intra- and interspecific crosses that generate fertile hybrids with the aim of releasing new cultivars. These aspects are covered in more detail in the following sections.

## Germplasm Resources

A germplasm bank is a living collection of the entire genetic heritage of a species. This collection aims to preserve the genetic variability of important crops and offers opportunities for research that characterize phenotypic and genetic variations (Migicovsky et al., 2019). Thus, the availability of and access to the germplasm resources of *Urochloa* spp. are vital for the development of new cultivars with superior agronomic and nutritional qualities, improved adaptation to environments, and greater resistance to pests and pathogens.

Throughout the world, seven important *Urochloa* germplasm collections are maintained in the field, mainly due to the difficulty in preserving the materials from the seeds. These collections constitute a huge gene pool, accounting for 987 accessions of 33 described species, and are located at the International Center for Tropical Agriculture – CIAT (601 accessions; Triviño et al., 2017), International Livestock Centre for Africa – ILCA (520 accessions; Keller-Grein et al., 1996), Brazilian Agricultural Research Corporation – Embrapa (455 accessions; Valle et al., 2008), Australian Tropical Forage Genetic Resource Center – ATFGRC/CSIRO (177 accessions), United States Department of Agriculture - USDA (90 accessions), National Genebank of Kenya – GBK (51 accessions), and Roodeplaat Pasteur Institute/African Research Council – RGI/ARC (39 accessions; Keller-Grein et al., 1996).

One of the largest germplasm banks is located at Embrapa Beef Cattle, Brazil, and is composed of 14 African species of *Urochloa* imported from the International Center for Tropical Agriculture (CIAT) in Cali, Colombia (Keller-Grein et al., 1996). These species of *Urochloa* include the four main species with the greatest agronomic value for use in pastures: *U. brizantha* (226 accessions), *U. decumbens* (52 accessions), *U. humidicola* (58 accessions), and *U. ruziziensis* (25 accessions).

The Embrapa collection has been evaluated in terms of morphological and agronomic characteristics, reproductive mode (apomictic or sexual), cytogenetics, and genetic diversity, this one mainly involving the use of molecular markers. (e.g., Valle and Savidan, 1996; Mendes-Bonato et al., 2002a,b; Boldrini et al., 2006; Risso-Pascotto et al., 2006;Nielen et al., 2009; Ambiel et al., 2010; Jungmann et al., 2010; Azevedo et al., 2011; Boldrini et al., 2011a; Vigna et al., 2011a; Garcia et al., 2013; Pessoa-Filho et al., 2015; Santos et al., 2015a; de Paula et al., 2017; Souza et al., 2018; Corrêa et al., 2020; Moraes et al., 2021). Based on morphological and agronomic traits, *U. brizantha* exhibited the greatest variation, which reflects the larger number of accessions and the greater inherent genetic variability. However, the characterization of morphological variability is limited due to the difficulty in characterizing some phenotypes as well as the phenotypic plasticity found in polyploids and species that form an agamic complex, such as *Urochloa* grasses. According to Bayer (1987), an agamic complex is represented by a group of species that includes sexual diploids and polyploids among facultative or obligate apomicts, which are largely the result of various hybridizations among sexual diploid and polyploid members.

Most of the accessions in the Embrapa collection are polyploid and apomictic with the exception of *U. ruziziensis*, which only includes diploid and sexual accessions (Valle and Savidan, 1996). Swenne et al. (1981) used colchicine to create sexual tetraploid *U. ruziziensis* genotypes that are important in interspecific crosses with tetraploid apomictic accessions of *U. decumbens* and *U. brizantha*. These tetraploidized genotypes became part of the basis of the breeding program at Embrapa and allowed release of the first commercial interspecific hybrid of *Urochloa* spp., which is known as BRS Ipyporã (*U. ruziziensis* x *U. brizantha*; Table 1). Additionally, through the use of colchicine, diploid sexual accessions of *U. decumbens* from the Embrapa collection have been duplicated (Simioni and Valle, 2009) and used in crossings within the species itself and with *U. brizantha* (Monteiro et al., 2016).

Cytogenetic characterization studies with this *Urochloa* collection started in 1998 at State University of Maringa. Cytogenetic analyses, such as the evaluation of chromosomal behavior in all phases of meiosis and counting the number of chromosomes, have already been performed with accessions of *U. brizantha* (Mendes-Bonato et al., 2002a; Risso-Pascotto et al., 2003a; Pagliarini et al., 2012), *U. decumbens* (Risso-Pascotto et al., 2002; Mendes-Bonato et al., 2002b; Junqueira-Filho et al., 2003; Ricci et al., 2010, 2011), *U. ruziziensis* (Risso-Pascotto et al., 2003b, 2005), and *U. humidicola* (Boldrini et al., 2006; Adamowski et al., 2007; Boldrini et al., 2011a,b), among other species. Cytogenetic analyses have also been performed with interspecific hybrids (Risso-Pascotto et al., 2004a,b; Mendes-Bonato et al., 2006, 2007; Adamowski et al., 2008; Felismino et al., 2010, 2012; Fuzinatto et al., 2012, 2017; da Rocha et al., 2019; de Campos Moraes et al., 2019) as well as intraspecific hybrids of *U. decumbens* (Souza, 2013). These cytogenetic studies are essential to selection of the best and most cytogenetically stable parents and hybrids in the direction of crosses (Simeão et al., 2021). As an example, Pagliarini et al. (2012) cytologically evaluated microsporogenesis in 46 accessions of *U. brizantha* from Embrapa Beef Cattle germplasm and found high percentage of meiotic abnormalities among accessions (ranging from 0.36 to 95.76%), which may affect pollen viability through generation of unbalanced gametes. Thus, the findings allowed selecting suitable genotypes as pollen donors for intra and interspecific crosses.

A part of the collection covering these five species has not yet been characterized by molecular markers, and this analysis is extremely necessary not only for obtaining knowledge of the genetic resources available but also for the development of core collections that represent, with a limited set of accessions, the diversity of the entire germplasm of *Urochloa* spp. (Frankel, 1984). Thus, rare alleles can be explored through genotypic combinations for both new intraspecific and interspecific crosses with the aim of developing heterotic cultivars for traits of interest in a more optimized manner *via* breeding programs.

In addition, the large-scale phenotyping of the core collection for different characteristics and the development of specific subsets for traits, such as spittlebug resistance and drought and heat tolerance, represents a singular opportunity to identify new alleles and develop new cultivars for breeding the next generation of *Urochloa* pastures.

## Genomic Tools and Resources

The molecular knowledge and the availability of genomic resources of *Urochloa* species used in pastures have only started to increase in the last decade. In this topic, we present the main studies and knowledge obtained about the four commercials *Urochloa* spp., using molecular and genomic tools. Briefly, we also expose some needs and perspectives for advancing genomic improvement in *Urochloa* spp.

### Genetic Diversity Assessed by Molecular Markers

The evaluation and characterization of the available genetic diversity between and within species are extremely important for conservation and genetic breeding. Data on the genetic diversity and population structure of *Urochloa* spp. obtained using molecular markers were reported just over 10 years ago (Table 3). The first studies were performed using random amplified polymorphic DNA (RAPD) markers (Chiari et al., 2008; Ambiel et al., 2010) and generated the first information about the genetic diversity present in *Urochloa* species at the molecular level.

**Table 3:** Main studies of the genetic diversity of *Urochloa* spp. using molecular markers.

Species	Molecular markers	N° of markers	References
*Urochloa* spp.	ISSR/RAPD/SSR	279*	Chiari et al. (2008); Ambiel et al. (2010); Almeida et al. (2011); Garcia et al. (2013); Nitthaisong et al. (2016); Ondabu et al. (2017); Triviño et al. (2017); Kuwi et al. (2018); Namazzi et al. (2020)
*U. humidicola*	SSR	27	Jungmann et al. (2010)
*U. brizantha*	SSR/ISSR	48*	Vigna et al. (2011a); Braga et al. (2017); Tegegn et al. (2019)
*U. ruziziensis*	SSR/ISSR	27*	Azevedo et al. (2011); Pessoa-Filho et al. (2015)

* *Sum of the number of markers in all cited studies*.

Shortly thereafter, other *Urochloa* studies began to be developed using another type of marker known as microsatellites or simple sequence repeats (SSRs; Tables 3, 4), which stand out for being multiallelic and more abundant in plant genomes than RAPD markers. SSRs are also more informative because they are codominant but need to be evaluated as dominant markers in *Urochloa* genotypes, as these species are predominantly polyploids. In this case, each band or allele of an SSR primer is considered a separate dominant marker, like in RAPD genotyping. Jungmann et al. (2010) used SSR markers to analyze the genetic diversity and population structure of 58 germplasm accessions and two cultivars of *U. humidicola* from Embrapa Beef Cattle. The accessions were highly structured into four major groups, and the coefficients of dissimilarity ranged from 0.02 to 0.89 considering all genotypes. The study observed that the phenotypic divergence of the only sexual accession of *U. humidicola* (H031) reflects a high genetic divergence because it comprises a mixture of distinct allelic pools not shared with most accessions (Jungmann et al., 2010). This considerable divergence between H031 and the other accessions of *U. humidicola* has also been observed in later studies and suggests a hybrid origin (Santos et al., 2015a; Vigna et al., 2016a).

**Table 4:** Microsatellite markers developed for *Urochloa* species.

Species	N° of polymorphic SSR markers developed	Transferability to other species	References
*U. brizantha*	28*	Yes	Jungmann et al. (2009a); Vigna et al. (2011a)
*U. decumbens*	116*	Yes	Ferreira et al. (2016); Souza et al. (2018)
*U. ruziziensis*	198	Yes	Silva et al. (2013);
*U. humidicola*	145*	Yes	Jungmann et al. (2009b); Santos et al. (2015a); Vigna et al. (2016a)

* *Sum of the number of markers in all cited studies*.

The genetic diversity and population structure of 172 germplasm accessions and six cultivars of *U. brizantha* were also evaluated using microsatellite markers (Vigna et al., 2011a). As a result, the genetic similarity among all genotypes ranged from 0.40 to 1.00, and two duplicates were found in the germplasm. Structural analyses have suggested germplasm separation into three clusters with significant differentiation among the various clusters (Vigna et al., 2011a). For *U. ruziziensis*, a study was developed to estimate the molecular diversity among 93 genotypes of Embrapa’s collection using inter-simple sequence repeat (ISSR) markers. The genetic similarity ranged from 0.0 to 0.95, revealing clones and highly divergent genotypes (Azevedo et al., 2011). Recently, the genetic diversity and population structure of 112 Ethiopian *U. brizantha* accessions (International Livestock Research Institute Forage Field Genebank) collected in nine different regions of Ethiopia and six *Urochloa* cultivars were analyzed using 23 SSR markers. The structural analysis revealed three clusters with distinct gene pools and significant variation among them. This study established a core collection of Ethiopian *U. brizantha* accessions consisting of 39 accessions (Tegegn et al., 2019).

The above-mentioned studies were performed within a single species of *Urochloa* genus, but studies of genetic diversity were also performed with more than one species. Using RAPD markers, Chiari et al. (2008) assessed the genetic diversity of four *Urochloa* spp. and found a genetic similarity ranging from 0.49 to 0.87 considering all genotypes analyzed. The greatest genetic dissimilarity was detected among genotypes of *U. ruziziensis* (0.73 to 0.83). In addition, the analysis showed that *U. brizantha*, *U. decumbens*, and *U. ruziziensis* were genetically closer to each other than to *U. humidicola*. This finding has also been observed in later studies using SSR markers (Jungmann et al., 2009a; Silva et al., 2013; Santos et al., 2015a; Ferreira et al., 2016; Triviño et al., 2017; Pessoa-Filho et al., 2017; Souza et al., 2018).

Nitthaisong et al. (2016) assessed the genetic diversity of 11 *Urochloa* species using 10 ISSR markers and found high polymorphism between the genotypes. The genotypes were grouped into three clusters: one cluster comprised the majority of polyploid plants (apomictic), and the other two clusters were mainly formed of diploid plants (sexual). *U. decumbens*, *U. brizantha*, *U. humidicola*, and *U. dictyoneura* were grouped into a separated cluster of *U. ruziziensis*. Using microsatellite markers to assess the genetic diversity among Kenyan *Urochloa* spp., Ondabu et al. (2017) found an average diversity of 0.623, with a range of 0.317 to 0.802. Triviño et al. (2017) also used SSR markers to analyze genotypes of *Urochloa* spp., which mainly originated from the CIAT germplasm, at the molecular level. In this study, the genotypes were distributed in four clusters, and one cluster was composed of most genotypes of *U. decumbens* and *U. ruziziensis*, as supported by morphological similarities between the two species (Renvoize et al., 1996). In addition, greater genetic variance was observed within the taxonomic groups (57%) than between groups (43%).

*Urochloa* genotypes from Tanzania were also evaluated in terms of their genetic diversity and population structure using SSR markers, and high genetic variation was found between the *Urochloa* genotypes. The population of *U. brizantha* showed the highest level of genetic diversity. All genotypes were divided into three main groups, and the genetic variation was greater within populations than among populations (Kuwi et al., 2018).

A national collection of *Urochloa* ecotypes from Uganda, possibly represented by different species, was evaluated using 24 SSR markers (Namazzi et al., 2020). These markers had a high discriminating ability with an average polymorphism information content (PIC) of 0.89 and detected 584 alleles in 99 ecotypes. A structural analysis showed the presence of three distinct gene pools in the Ugandan *Urochloa* ecotypes, in agreement with previous studies performed in Brazil, Tanzania and Ethiopia (Vigna et al., 2011a; Kuwi et al., 2018; Tegegn et al., 2019).

These studies revealed significant genetic diversity between and within the main *Urochloa* species, and this finding has also been observed phenotypically. Hundreds of SSR markers are available specifically for *Urochloa* species, and many of these markers are transferable to different species (Jungmann et al., 2009a,b; Vigna et al., 2011b; Silva et al., 2013; Santos et al., 2015a; Ferreira et al., 2016; Souza et al., 2018; Table 4). Therefore, further studies must be carried out with the available molecular markers, mainly involving characterization of the entire germplasms. For instance, the molecular characterization of *U. decumbens* germplasm accessions still needs to be performed. In addition, molecular studies to characterize the diversity and genetic structure of *Urochloa* spp. germplasm and hybrids can be performed rapidly and effectively using the latest genotyping technologies, including genotyping-by-sequencing (GBS).

### Molecular Phylogenetics

The grass family (Poaceae) comprises subfamilies that are divided into two large clades: the BOP (Bambusoideae, Oryzoideae, and Pooideae) clade and the PACMAD (Panicoideae, Aristidoideae, Chloridoideae, Micrairoideae, Arundinoideae, Danthonioideae) clade (Kellogg, 2015, Soreng et al., 2015, Soreng et al., 2017, Saarela et al., 2017). The subfamily Panicoideae (approximately 3,500 species in 12 tribes; Bhatt and Thaker, 2021) forms a monophyletic group that includes, in addition to *Urochloa*, other important economic species, such as maize (*Zea mays*), sugarcane (*Saccharum officinarum*) and sorghum (*Sorghum bicolor*; Hodkinson, 2018). This monophyly has been supported by several molecular phylogenetic studies (Vicentini et al., 2008; Sánchez-Ken and Clark, 2010), including chloroplast and nuclear gene analyses, that aimed to establish a robust phylogenetic framework for unraveling the evolution of this ecologically and economically important subfamily (Kellogg, 1988; Soreng and Davis, 1998; Bhatt and Thaker, 2021).

Giussani et al. (2001) analyzed the molecular phylogeny of the subfamily Panicoideae using sequencing data from the chloroplast gene *ndhF*. Based on this study, the species were divided into three strongly supported clades, and species of the *Urochloa* genus were included in the Paniceae clade (base chromosome number of *x* = 9) together with the *Setaria* species and most of the *Panicum* and *Pennisetum* species. This study showed that *Urochloa*, *Setaria*, and *Pennisetum* are paraphyletic genera. Additionally, in Panicea, Kellogg (2015) placed *Megathyrsus* in *Urochloa*, and Washburn et al. (2015) found that the *Urochloa* and *Digitaria* genera are paraphyletic.

*Urochloa* belongs to the Melinidinae subtribe, which is monophyletic (Giussani et al., 2001; Vicentini et al., 2008; Salariato et al., 2010). A chloroplast phylogenetic study of this subtribe discussed the paraphyly of *Urochloa* (Salariato et al., 2009). In addition, a molecular phylogenetic tree found two major clades comprising species of the Melinidinae subtribe, and all *Urochloa* species were placed in clade II with some species of *Eriochloa* as well as *Chaetium*, *Megathyrsus* and *Scutachne* (Salariato et al., 2010).

Recently, genomic datasets (particularly plastomes) have been evaluated to investigate the evolutionary relationships in several grass subfamilies, including Panicoideae (Jones et al., 2014; Cotton et al., 2015; Burke et al., 2016; Saarela et al., 2018; Duvall et al., 2019; Orton et al., 2021), and have contributed to greater resolution of and support for relationships within and among grass subfamilies (Saarela et al., 2018). Based on transcriptome-based phylogenetic inference, Washburn et al. (2017) revealed a Paniceae nuclear species tree in which the Anthephorinae subtribe is a direct sister to the MPC clade (subtribes Melinidinae, Panicinae, and Cenchrinae), corroborating the findings reported by Vicentini et al. (2008). In this phylogenetic tree, *U. brizantha* was placed together with *U. plantaginea*, and both in the same clade that *U. fusca* and *Megathyrsus maximus*, another important tropical forage grass used in pastures.

The phylogenetic studies conducted specifically with *Urochloa* remain limited. Torres González and Morton (2005) analyzed 22 genotypes of different species of the *Brachiaria* and *Urochloa* genera using ribosomal internal transcribed spacer (ITS) sequences as the molecular dataset and eight morphological spikelet characters (out of 17) used by the informal grouping reported by Renvoize et al. (1996). The main objective was to analyze the phylogenetic relationships of these genera. These researchers observed that *B. humidicola* and *B. dictyoneura* were grouped in the same clade, whereas *B. ruziziensis* and *B. brizantha* were placed together in a different clade than *B. decumbens*. No relationship was detected between morphological traits and molecular results. In addition, the authors classified *Brachiaria* as a monophyletic complex within the genus *Urochloa*.

Using chloroplast DNA (cpDNA), Salariato et al. (2009) found that *U. decumbens* and *U. brizantha* were closely related and separate from *U. humidicola* and *U. dictyoneura*. Similarly, *U. ruziziensis*, *U. brizantha*, and *U. decumbens* were included in a strongly supported clade separate from *U. humidicola*, corroborating the results from other molecular studies. Therefore, these results were different from those found by Torres González and Morton (2005), who included *U. decumbens* in another group based on molecular and morphological data.

Pessoa-Filho et al. (2017) evaluated the phylogenetic divergence between *Urochloa* species based on complete chloroplast genomes and observed that most of the polymorphisms between the species were located in intergenic regions, reflecting their phylogenetic distances. The phylogenetic analysis of this study yielded well-supported clades, and the results corroborated the findings reported by Salariato et al. (2009, 2010): *U. humidicola* is a sister taxon of the *U. ruziziensis*, *U. brizantha*, and *U. decumbens* clades. Also, *U. decumbens* and *U. brizantha* chloroplast sequences were highly similar and the phylogenetic tree showed that these species are phylogenetically closer to each other compared to *U. ruziziensis* and *U. humidicola*. The above-mentioned study is the only analysis of complete chloroplast genome sequences of *Urochloa* species and generated data useful for genetic analysis.

Future phylogenetic studies with *Urochloa* species need to include more samples of each species and with different ploidies to provide better representation and thereby improve the understanding of the phylogenetic relationships between these species. With advances in genomics, new tools and resources are available to fill the gaps in the phylogenetic characterization of the *Urochloa* genus, including next-generation sequencing (NGS) data and bioinformatics methods. This advance has transformed molecular phylogenetics into phylogenomics (Mardis, 2011; Young and Gillung, 2019). As an example, GBS and RADseq (restriction site-associated DNA sequencing) are being used for phylogenomics analyses of important plant species (Girma et al., 2018; Blanco-Pastor et al., 2019), and RNA sequencing (RNA-Seq) is being used for the development of phylogenetic markers, including SSR and targeted enrichment (Vatanparast et al., 2018; Liu et al., 2021).

### Cytogenetics

The field of cytogenetics addresses chromosomes and their inheritance, including their function, movement, numbers and structure. In addition, this field includes modifying the structure and behavior of chromosomes in relation to processes such as the recombination, transmission and expression of genes (Singh, 1993).

*Urochloa* species have various ploidy levels, ranging from diploid to nonaploid (Table 2), and this variation is characterized by a predominance of polyploidy, particularly tetraploidy (Valle and Pagliarini, 2009). The basic number of chromosomes was established by Darlington and Wylie (1955) as *x* = 7 or *x* = 9, with a predominance of the latter (Bernini and Marin-Morales, 2001; Valle and Pagliarini, 2009). However, cytological studies performed with *U. dictyoneura* and *U. humidicola* showed evidence for *x* = 6 as the base chromosome number for these species because of the presence of hexavalents in accessions with 2n = 36 and 2n = 42 chromosomes and octa- and nonavalents in accessions with 2n = 54 chromosomes (Risso-Pascotto et al., 2006; Boldrini et al., 2009).

Regarding the chromosome number, the highest value (2n = 90) was reported in *U. bovonei* (Spies and Du Plessis, 1986). For *U. brizantha*, *U. decumbens*, and *U. humidicola*, some studies have described plants with different numbers of chromosomes depending on the level of ploidy (Basappa et al., 1987; Bernini and Marin-Morales, 2001; Valle and Pagliarini, 2009; Jungmann et al., 2010). For *U. ruziziensis*, a diploid species, cytogenetic descriptions have shown 2n = 2x = 18 chromosomes (Bernini and Marin-Morales, 2001), whereas artificially induced tetraploid plants have 2n = 36 chromosomes (Timbó et al., 2014a).

Duplication of the chromosome number using colchicine was achieved for the first time in genotypes of *U. ruziziensis* (Gobbe et al., 1981; Swenne et al., 1981), later in diploid accessions of *U. brizantha* (Pinheiro et al., 2000) and *U. decumbens* (Simioni and Valle, 2009) and again in *U. ruziziensis* (Ishigaki et al., 2009; Timbó et al., 2014b). Artificial tetraploid plants have been important for the realization of inter- and intracrosses in breeding programs and thus allow exploration of the genetic variability maintained by apomixis (Felismino et al., 2010).

Chromosome research, particularly meiotic analysis, has been performed with *Urochloa* species and hybrids (Mendes-Bonato et al., 2002a,b, 2006, 2007; Risso-Pascotto et al., 2002, 2003a,b, 2004a,b, 2005; Junqueira-Filho et al., 2003; Boldrini et al., 2006, 2009, 2011a,b; Adamowski et al., 2007, 2008; Felismino et al., 2010, 2012; Ricci et al., 2010, 2011; Fuzinatto et al., 2012, 2017; Pagliarini et al., 2012; Souza, 2013). These studies have revealed several abnormalities typical of allopolyploids and segmental allopolyploids, such as asynchronous chromosome segregation, intragenomic pairing, and the presence of multivalents and micronuclei. More recently, de Paula et al. (2017) confirmed the allopolyploidy of *U. decumbens* 4x and *U. brizanth*a 4x and Corrêa et al. (2020) observed that the diploid genotypes of these species are potential ancestors of allotetraploids. Both studies were performed *via* genomic *in situ* hybridization (GISH).

Karyotypes of *Urochloa* species were constructed by Bernini and Marin-Morales (2001), who were the first to describe the variations in the relative chromosome length and satellite position among accessions of *Urochloa*. These differences may be indicative of the coexistence of different genomes or chromosomal rearrangements in the species. Akiyama et al. (2010) reported differences in the number and morphology of ribosomal DNA (rDNA) sites in the accessions of *U. brizantha*, *U. ruziziensis*, *U. humidicola*, and the *Urochloa* hybrid Basappa & Muniy. cultivar Mulato (originated from the crossing between *U. decumbens* and *U. ruziziensis*). More recently, Moraes et al. (2021) characterized the karyotype of a pentaploid cytotype of *U. brizantha* by the mapping of rDNA sites and nuclear DNA content analysis. The karyotype organization that has been observed suggests an allopolyploid origin for *U. brizantha*, corroborating the results from a study of the physical mapping of rDNA genes in this species (Nielen et al., 2009). In another study, the occurrence, mapping, and distribution of gypsy retrotransposons in *U. decumbens*, *U. brizantha*, *U. ruziziensis* and *U. humidicola* showed that mobile elements, in addition to the chromosome number variation, contributed to karyotype evolution (Santos et al., 2015b; Worthington et al., 2021).

Although cytogenetic studies of the *Urochloa* genus are in progress, there remain many gaps to be filled. For example, the cytogenetic map of the genus is still incomplete, mainly due to the small size and morphological similarity of chromosomes (Nani et al., 2018). Again, the “era of omics” and computational advances can influence and expand the cytogenetics-related knowledge of *Urochloa* spp. For instance, oligo- and CRISPR-FISH have been used to improve our understanding of the karyotype and chromosomal variation and evolution in important crops, including polyploid species (Braz et al., 2017; Liu et al., 2019; Li et al., 2020). Chromosome-scale assembly based on single-chromosome optical mapping is also a recently developed approach for analyzing structural variations in large regions (Deschamps et al., 2018) that can provide important insights into genetic variation and for guiding genome assemblies (Leinonen and Salmela, 2020).

### Genetic Mapping and Quantitative Trait Locus Detection

Genetic maps are models that describe the distances and relative positions of a group of genes or markers along the chromosomes of a given species. These maps estimate the recombination frequencies, and this estimation provides information about the inheritance of loci that control characteristics of interest (Bourke et al., 2018a). For cases in which the genome has not yet been fully sequenced, as is the case for most species of the genus *Urochloa* spp., genetic maps are valuable tools for understanding the genetic and genomic organization of species (Aitken et al., 2014). Even for species with a sequenced genome, searching for QTLs remains a strategy for maintaining a relationship between the phenotype and the genotype (Lewin et al., 2009). For example, a QTL found in a biparental population identifies genomic regions for which the parents are polymorphic and whose variation can be associated with phenotypic variation, which suggests a link between the genes and the trait in question (Thérèse Navarro et al., 2020).

In the case of polyploid species, as are most of the species of the genus *Urochloa* spp., the challenge in the construction of genetic maps is greater than that found with diploid species due to a greater number of allele combinations and the markedly higher number of possible genotypes in a segregating progeny (Bourke et al., 2018a; Mollinari and Garcia, 2019; Mollinari et al., 2020). In addition, the type of polyploidy is often not well defined (autopolyploid or segmental allopolyploid), which makes it difficult to determine the expected patterns of segregation assumed during the construction of a genetic map (Bourke et al., 2018a,b). These and other reasons, such as self-incompatibility and high levels of heterozygosity, explain the difficulty in building high-resolution genetic maps for *Urochloa* spp. Consequently, few genetic maps have been developed (Thaikua et al., 2016; Vigna et al., 2016a; Worthington et al., 2016, 2019; Ferreira et al., 2019; Worthington et al., 2021; Table 5).

**Table 5:** Genetic mapping studies of *Urochloa* species.

Species	Molecular marker	N° of molecular marker	References
*U. humidicola*	SSR	102	Vigna et al. (2016a)
*Urochloa* spp.	SNP, SCAR	1,916	Worthington et al. (2016)
*Urochloa* spp.	AFLP	272	Thaikua et al. (2016)
*U. decumbens*	SNP	1,000	Ferreira et al. (2016)
*U. humidicola*	AFLP, SSR, SNP, KASP, InDel	4,210	Worthington et al. (2019)
*Urochloa* spp.	SNP, InDel	4,427	Worthington et al. (2021)

*SSR, simple sequence repeat; SCAR, sequence characterized amplified region; AFLP, amplified fragment length polymorphism; SNP, single nucleotide polymorphism; KASP, kompetitive allele specific PCR; InDel, insertion–deletion*.

In the first study, Thaikua et al. (2016) used the approach of utilizing separate parents to construct an amplified fragment length polymorphism (AFLP)-based linkage map of the apomictic cultivar “Mulato” (Miles et al., 2004). This cultivar was crossed with three plants of sexual *U. ruziziensis* cv. “Miyaokikoku,” which generated 84 hybrids. The linkage map had a total length of 1423.2 cM, with 272 AFLP markers, and the apomixis locus was assigned to linkage group 2. Additionally, QTLs associated with agriculturally important traits (morphological traits and percentage of filled seeds) were identified by simple interval mapping (SIM) and composite interval mapping (CIM).

Worthington et al. (2016) constructed the first genetic map based on SNP markers for *Urochloa* spp. from 167 hybrids generated from a cross between the maternal synthetic autotetraploid *U. ruziziensis* (BRX 44–0) and the paternal *U. decumbens* cv. Basilisk (CIAT 606). Separate genetic linkage maps were constructed for each parent following the two-way pseudo testcross strategy. For the maternal linkage map, 706 SNP markers were placed in 34 linkage groups, with a total length of 1985 cM. In contrast, 1,210 SNP markers and markers associated with apomixis were assigned to 36 linkage groups in the CIAT 606 genotype, with a total length of 2,693 cM. These maps were used to assess synteny with foxtail millet (*Setaria italica* (L.) P. Beauv), the closest relative of *Urochloa* spp. with a publicly available reference chromosome-scale genome. Synteny was highly conserved between *U. decumbens*, *U. ruziziensis*, and foxtail millet, with only one major structural rearrangement. This provides evidence for the quality of the genetic maps, enabling the assignment of linkage groups to chromosomes and the identification of homologs. The “apospory-specific genomic region” (ASGR) was mapped to position 42.5 cM of group 5c, a region syntenic with foxtail millet chromosome 5 (Zhang et al., 2012).

Using SSR markers, Vigna et al. (2016a) built the first linkage map to investigate apomixis in the hexaploid *U. humidicola*. A full-sib F_1_ population obtained by crossing the sexual accession H031 (CIAT 26146) and the apomictic cultivar *U. humidicola* cv. BRS Tupi was used to generate an integrated genetic map *via* the multipoint approach based on Markov chains. Forty-nine linkage groups (LGs) were created, with a total length of 1702.82 cM. Eight homology groups (HGs) were formed, and the apo-locus that controls apospory was mapped in LG02 (Pessino et al., 1997, 2004).

More recently, another linkage map was obtained for *U. humidicola* using SNP, AFLP, SSR, insertion–deletion (InDel) and kompetitive allele specific PCR (KASP) markers (Worthington et al., 2019). The sexual accession CIAT 26146 [EMBRAPA Beef Cattle (EBC) H031] was crossed to the apomictic male parent CIAT 16888 (EBC H027), generating 124 hybrids that were used to construct separate parental linkage maps following the two-way pseudo-testcross strategy. As a result, two haplotype maps (each with 36 linkage groups) were generated. The maternal haplotype map had 2,589 markers and a length of 3,558 cM, and the paternal haplotype map had 1,621 markers and 4,363 cM. Additionally, the ASGR, which is known to be a single dominant Mendelian factor of apomixis in Paniceae grasses (Ozias-Akins and van Dijk, 2007), was mapped to position 55.8 cM of CIAT 16888 (paternal haplotype) linkage group 1b. Synteny analysis of the *U. humidicola* parental maps with the foxtail millet physical map supported the previous cytogenetic evidence for a base chromosome number of x = 6 in the species (Boldrini et al., 2011a). The authors observed a high degree of synteny between some chromosomes of *U. humidicola* and foxtail millet, *U. decumbens* and *U. ruziziensis*. In addition, they accounted for the fusion of three pairs of chromosomes, which is consistent with the large chromosome size of *U. humidicola* relative to other *Urochloa* species (Bernini and Marin-Morales (2001)).

Additionally, in 2019, Ferreira et al. developed the first intraspecific genetic map of *U. decumbens*. Crossbreeding was performed between sexual *U. decumbens* D24/27 (artificially tetraploidized with colchicine) and apomictic *U. decumbens* cv. Basilisk, and 217 hybrids were selected. This mapping population was used to construct a consensus linkage map using the additional information provided by the SNP allele dosages in contrast to traditional methods based on allele presence/absence data (Hackett et al., 2013). A total of 1,000 SNP markers were distributed throughout nine homology groups, with a cumulative length of 1,335.09 cM. In addition, the genetic map allowed the identification of genomic regions related to resistance to spittlebug (*Notozulia entreriana* Berg), which is the main pest insect that attacks *U. decumbens*. The percentages of phenotypic variation explained by the QTLs ranged from 4.66 to 6.24%, reflecting the median heritability ( $H_{C}^{2}=0.37$ ) found for spittlebug resistance in the population.

Recently, a new genetic map was constructed for the same interspecific progeny of Worthington et al. (2016), composed for 169 hybrids generated from a cross between a synthetic autotetraploid accession of *U. ruziziensis* (BRX 44–02) and *U. decumbens* cv. Basilisk (CIAT 606), used as male parent (Worthington et al., 2021). The analyses were based on the genetic map of the male parent, which included 4,427 markers distributed in 18 linkage groups, as expected for the segmental allotetraploid *U. decumbens* (2*n* = 4*x* = 36). Once again, the authors observed synteny between *U. ruziziensis* and foxtail millet, with three large translocations between chromosomes 1 and 7, 2 and 6, as well as 3 and 5, and four inversions between tails in chromosomes 1 and 4, 2 and 9, and 2 and 3. This map allowed the mapping of three significant QTLs associated with root length, biomass under both control and Al3+ stress conditions, and root diameter under stress/control conditions, which explained 12.8 to 16.1% of the phenotypic variance (Worthington et al., 2021).

Genetic mapping is a widely used resource to identify genomic regions responsible for important agronomic traits. One of the first steps in MAS is the development of genetic markers and high-resolution linkage maps, and this step is followed by the mapping of loci related to traits of agronomic and commercial importance. However, despite efforts aimed at building genetic maps for species of *Urochloa* spp., there is a clear need to improve the quality and increase the resolution of these maps, to map target genomic regions, and to build new maps for other species, including *U. brizantha*. Recently, packages capable to use all possible segregations for autopolyploid and segmental allopolyploid species were implemented in the R software (polymapR - Bourke et al., 2018b; MAPpoly - Mollinari and Garcia, 2019; Mollinari et al., 2020; R Core Team, 2020). These advances may enable the construction of integrated linkage maps with high resolution in polyploid species of *Urochloa* spp.

In addition, the reference genomes currently available (Pessoa-Filho et al., 2019; Worthington et al., 2021), new statistical and computational resources, and the possibility of large-scale genotyping followed by estimation of multiple dosages of SNP loci may aid the development of genetic maps for *Urochloa* spp. with high density and resolution (Bourke et al., 2018a). Although these tools do not have an immediate impact on breeding, the scanning of important genetic information from the genome for genes related to target traits provides a crucial basis for MAS, in addition to promoting faster and more effective molecular improvements of *Urochloa* species.

### Genome-Wide Association Studies

Although genetic maps of biparental populations provide a framework for detecting rarely occurring alleles that have a large effect on the phenotype in breeding populations, GWASs, also known as association mapping studies, reflect historical recombination events in diversity panels and are an efficient approach for locating common alleles associated with phenotypes with higher mapping resolution than that achieved with linkage mapping. Together, linkage mapping and GWAS can detect QTLs and elucidate the genetic basis of important traits more efficiently (Talukder et al., 2019).

Similar to genetic maps, GWASs have also benefited from high-throughput marker systems, which allow the detection of markers rapidly and at low cost. However, associative mapping studies of polyploid species remain extremely recent, and only one such study has investigated the *Urochloa* genus (Matias et al., 2019a). This study was performed with a set of 263 tetraploid interspecific *Urochloa* hybrids using 26,535 SNP markers with different allele dosage configurations obtained by GBS. Also were evaluated scenarios including additive, dominance, and epistatic effects. Associations were studied regarding agronomic and nutritional forage traits, including the regrowth capacity and crude protein. Seven SNP markers associated with the main forage traits were identified and these allow a better understanding of the *Urochloa* genome for important economic traits. Additionally, these markers can be useful to speed up breeding program selection cycles.

The authors observed that it is possible to identify the same genomic regions using diploid and tetraploid models, although in some cases the allele substitution effect can be biased. Then, the authors suggest the use of markers with both diploid and tetraploid molecular configurations to account for all regions of the genome in allopolyploid species (Matias et al., 2019a).

This study opens new perspectives for understanding the inheritance of complex traits in *Urochloa* species and segmental allopolyploids in general. GWASs constitute a promising tool for the identification of molecular markers associated with characteristics of interest that can be used in MAS to accelerate breeding cycles. In the genetic improvement of *Urochloa* species, in addition to enabling the identification of markers associated with agronomic and nutritional characteristics, GWASs are an important resource in the search for markers linked to resistance to insect pests, such as spittlebugs, and other biotic and abiotic stresses that influence forage production, which are characteristics that were identified as research priorities in the next-generation breeding of tropical forages in Brazil by Pereira et al. (2018).

### Genomic Selection and Prediction

Genomic selection (GS), which was proposed by Meuwissen et al. (2001), is a powerful and modern breeding tool for identifying and exploiting superior genotypes based on the use of thousands of distributed markers across the genome to predict genetic values (Bhat et al., 2016). The improvement in the accuracy of the selected individuals and the reduction in the duration of the selection cycle time reflect the acceleration of genetic gains achieved by GS in breeding programs. At present, the selection process until the release of new perennial tropical forage cultivars takes approximately 10–15 years (Jank et al., 2014; Matias et al., 2019b). Because the genetic gains in yield achieved from the breeding of forage crops based on traditional methods of phenotypic selection are low (Casler and Brummer, 2008), GS has emerged as a potential opportunity to obtain significant gains in target traits and to reduce breeding cycles (Simeão-Resende et al., 2013; Simeão et al., 2021).

The incorporation of GS in breeding programs depends on several factors, such as the reproductive mode of the species, the genetic architecture and heritability of the characteristics of interest, and the total financial budget of the program, because large phenotyped and genotyped populations are necessary to estimate the effects of molecular markers (Yadav et al., 2020). In the case of tropical forage grasses, appropriately sized training populations, large panels of SNP markers and polyploidy are also important factors to be considered because these make GS even more challenging (Simeão et al., 2021). Therefore, although this potential tool has been widely applied in many species of economic importance, it has not yet been used in breeding programs of tropical forage grasses of the *Urochloa* genus (Simeão-Resende et al., 2013; Pereira et al., 2018; Simeão et al., 2021). Consequently, there is limited information regarding the application of genomic prediction and GS in tropical forage grasses compared with other important crops.

Simeão-Resende et al. (2013) performed a simulation study to compare the gain and accuracy of different GS methods considering the experimental plot designs used in forage breeding. This study concluded that the application of GS methods in tropical forage breeding has potential when the phenotypic evaluation of individual plants is incapable of predicting performance under sward conditions, when within-family selection pressure is difficult or impossible, and/or when phenotypic evaluations require long cycle times.

Using real data from a panel of interspecific hybrids genotyped by GBS, Matias et al. (2019b) evaluated the influence of multiple traits and allele dosage information on the genomic prediction accuracy in *Urochloa* spp. The authors concluded that genomic prediction should be used in *Urochloa* spp. genetic improvement with the aim of reducing time and costs because all models of GS evaluated provided greater genetic gains than phenotypic selection. In addition, SNP markers with allele dosages associated with additive, dominant, and multitrait factors have increased the accuracy of genomic prediction models.

A study of GS performed with *Megathyrsus maximus* (syn. *Panicum maximum*), which is another important polyploid forage grass used as pasture in tropical areas, resulted in low genomic prediction accuracies for traits such as leaf dry matter and regrowth capacity (de C Lara et al., 2019). Low values of prediction accuracy were also observed by Matias et al. (2019b), although GS allowed greater genetic gains than those obtained with phenotypic selection. Due to the polyploidy and high levels of heterozygosity of tropical forage grasses, there is a need to develop more accurate predictive models for the incorporation of GS in breeding schemes.

Advances in high-throughput technologies can influence the efficient use of GS in *Urochloa* breeding schemes with increases in the number and density of markers and large-scale phenotyping. Additionally, statistical and computational methods, including machine learning (ML) and simulation studies, can aid the development of predictive models most suitable for polyploids and the identification of the best potential GS-based breeding scheme designs, which would result in genetic gains with greater accuracy and shorter breeding cycles (Montesinos-López et al., 2021; Simeão et al., 2021).

### Transcriptome Profiles

The transcriptome is the set of RNA transcripts contained in a cell or tissue at a specific stage of development and/or under specific physiological conditions (Ozsolak and Milos, 2010). In this context, transcriptome studies allow identification of the molecular constituents of cells and tissues and understanding the functional elements of the genome and the molecular mechanisms involved in each condition, such as biotic stress in plants (Wang et al., 2009). The most effective approach for creating transcriptome profiles is RNA-Seq, which is performed using deep sequencing technologies (Wang et al., 2009; Kukurba and Montgomery, 2015).

Studies of the transcriptome of the *Urochloa* genus remain limited (Table 6). The first RNA-Seq study was performed with leaves from two genotypes of *U. humidicola* that are highly divergent in terms of phenotype and reproductive modes: the only sexual accession of the *U. humidicola* germplasm, known as BH031, and the apomictic cultivar BRS Tupi (Vigna et al., 2016b). The main objectives were to describe and compare the leaf transcriptome of these divergent genotypes, which were collected at the same field condition. As a result, approximately 76,000 transcripts were *de novo* assembled, which totaled 35,093 nonredundant unigenes. Most of these transcripts were annotated in the Phytozome database (Goodstein et al., 2011). From these unigenes, new EST-SSRs (4,489) and SNP markers (560,298) that may be associated with important traits for forage breeding, such as flood stress tolerance and the C4 pathway, were identified. Moreover, as in previous studies, a significant difference was observed between the analyzed genotypes, but now in relation to gene expression.

**Table 6:** Details of transcriptome sequences of *Urochloa* species.

Species	Tissue sample	Generated data (Gb)	Platform	Data repository	Reference
*U. humidicola*	Leaf	13.09	Illumina GAIIx	NCBI SRP065020	Vigna et al. (2016b)
*U. decumbens*	Root	NA	Illumina HiSeq 2000	NCBI SRP071168	Salgado et al. (2017)
*U. decumbens* and *U. ruziziensis*	Root and stem	NA	Illumina HiSeq 2,500	NA	Worthington et al. (2021)
Interspecific hybrids of *U. ruziziensis* and *U. decumbens*	Root and leaf	NA	Illumina HiSeq 2000	ENA PRJEB41722	Jones et al. (2021)

*NA, not available; ENA, European Nucleotide Archive; NCBI, National Center for Biotechnology Information*.

The next transcriptome of the genus *Urochloa* was assembled *de novo* from root sequences of *U. decumbens* cv. Basilisk under two controlled conditions: hydroponic solutions with and without aluminum (AlCl_3_). The assembly generated 164,930 transcripts and approximately 69% of these transcripts were attributed to a putative function of the protein *via* comparison with different protein databases. In addition, 13,375 microsatellite markers were identified in the transcripts. Differential expression analysis revealed a great difference in plants exposed to Al stress, and it was possible to identify transcripts with putative functions related to resistance and aluminum exposure. Therefore, this transcriptome represents a great resource for aiding the elucidation of the main molecular mechanisms of aluminum tolerance in forage grass species (Salgado et al., 2017).

Another transcriptome of the *Urochloa* genus was also assembled to investigate the molecular basis of toxic aluminum tolerance because this condition directly affects forage productivity and is common in tropical soils. Worthington et al. (2021) sequenced root and stem tissue samples of two replicated plants of *U. decumbens* CIAT 606 (cv. Basilisk) and *U. ruziziensis* BRX 44–02 under two conditions: growth in a high aluminum concentration (200 μM AlCl_3_) and under control conditions (0 μM AlCl_3_). As a result, most of the differentially expressed (DE) genes were regulated at the root of *U. decumbens*, which is a genotype classified as tolerant to aluminum. The authors identified different genes related to the stress response to aluminum, and the evidence shows that the tolerance mechanisms found in *Urochloa* are the same as those that occur in rice, another grass species.

The most recent transcriptome of the *Urochloa* genus investigated the transcriptomic profiles of leaves and roots of three *Urochloa* interspecific hybrid genotypes with the onset of water stress (Jones et al., 2021). According to a previous analysis, the genotypes were characterized as exhibiting good (gt-17), intermediate (gt-9) and poor tolerance (gt-18) to drought. As a result, the genotypes with good and intermediate tolerance to drought showed enriched DE genes that were more similar to each other. In addition, DE genes related to carbohydrate and cell wall metabolism in the leaves were upregulated in gt-9 and gt-18 and downregulated in gt-17. Because the level of water stress increased for all evaluated genotypes, an excess of downregulated putative apoplastic peroxidases was detected in the roots, which suggests that changes in the architecture of the root cell wall occur in response to the stress. As tolerance to abiotic stresses is one of the main objectives of *Urochloa* breeding, this study generated important information and knowledge about genes and metabolic pathways involved in the response to water stress (Jones et al., 2021).

Although these transcriptomes represent only a part of the transcripts of three species of *Urochloa*, they constitute important contributions to the scientific community interested in forage grass and breeding programs. The results provide relevant information on the genomic resources available for *Urochloa* grasses, mainly due to the possibility of identifying new candidate genes potentially related to characteristics of economic interest. These candidate genes can be further investigated by coexpression networks, which provide valuable information on the biological relationships between genes.

Even so, each species of *Urochloa* has its own peculiarities, and other transcriptome studies in which a specific stage of development and/or a physiological condition is considered must be performed. For instance, genetic information for some important characteristics, such as resistance to spittlebugs, apomictic reproduction, and flood tolerance, is still lacking, and transcriptomic studies are promising for generating new knowledge about these characteristics. The elucidation of different strategies of spittlebug resistance, such as information about the possible genes involved in this resistance and molecular markers in these genes, provides powerful information for breeding strategies and future studies, including GWAS and GS.

### Genome Sequencing

NGS has allowed genome sequencing to be a reality not only for model species but also for nonmodel or “orphan” species and polyploid organisms, such as *Urochloa* spp. However, despite this greater accessibility, the different levels of ploidy and different reproductive modes, in addition to the availability of different species with specific characteristics, are obstacles to obtaining a representative genome for *Urochloa* pastures.

The fact that breeding programs were recently created and the lack of financial resources (Pereira et al., 2018) also directly influence the availability of fundamental genomic resources. The existence of only two sequenced genomes representing these pasture grasses is a reflection of the above-described finding (Pessoa-Filho et al., 2019; Worthington et al., 2021). The first, the full characterization of which is not yet available, is a diploid chromosome-scale genome of a heterozygous clone of *U. ruziziensis*, named C6. Sequencing with PacBio Sequel, based in SMRT (Single Molecule Real Time) technology, generated more than 13.3 million long reads (~142x coverage) and allowed assembly of a genome covering 98.2% of the estimated haploid genome size of 615 Mbp for ruzigrass. Most of the assembled genome (94%) was included in nine scaffolds that correspond to the nine chromosomes of *U. ruziziensis*, with an N50 of 66 Mpb. Other metrics regarding genome completeness were satisfactory and included an NG50 of 286 kbp, 95.2% BUSCO (Benchmarking Universal Single-Copy Orthologs) complete matches and 83.3% complete single copies (Pessoa-Filho et al., 2019).

Additionally, using a diploid *U. ruziziensis* genotype (CIAT 26162) as a DNA source, a whole-genome assembly (WGA) was obtained by sequencing with short reads (approximately 100 × coverage) and presented satisfactory completeness (Worthington et al., 2021). The repetition content representing 51% of the total genome and the large number of Gypsy and copia long terminal repeats (LTRs) found were similar to those observed in foxtail millet (*Setaria italica* (L.) P. Beauv; Zhang et al., 2012), a species that is widely used as a reference in genomic studies of tropical forage grasses. Moreover, approximately 42,000 coding genes have been annotated, and most of these genes encode a protein showing homology to a protein in *S. italica*.

Worthington et al. (2021) prioritized the assembly of a diploid genome rather than a tetraploid genome because *Urochloa* grasses are heterozygous outcrossing species. Even so, a polyploid genome is essential for better representing the main accesses used for breeding and reflecting their genetic information, particularly the heterozygosity present. The recent emergence of third-generation sequencing (TGS) technologies can aid the complex and challenging assembly of these polyploid plant genomes because it produces high-quality genome assemblies with high resolution due to the longer length of the reads (Kyriakidou et al., 2018). Consequently, more genomic information is provided, and more genomic variants will be identified to aid genomic studies, such as those that address the genotype–phenotype–environment relationship, which will provide a great resource for improving *Urochloa* breeding programs.

We also emphasize that these available genomes, despite being diploid, represent an invaluable resource for orphan species because they aid the development and application of genomic tools for breeding and genetic studies of *Urochloa* grasses (Tang, 2017; Kyriakidou et al., 2018). Until then, our understanding of genomic organization was based on genetic maps, but it is now possible to go even further. The analyses that require the identification of variants, such as GS, GWAS, and genetic mapping, will benefit greatly from this genome because the use of genomes of species genetically close to *Urochloa* spp. as a reference is associated with a substantial loss of data. Therefore, exclusive information on *Urochloa* grasses may be revealed, and new approaches can be devised now that this fundamental genomic resource is available.

## Polyploid and Apomixis

The different ploidy levels and the apomixis represent reproductive barriers within and between species of the *Urochloa* genus, and these barriers challenge the genetic improvement of these grasses, which usually aims to produce new cultivars *via* intra- and interspecific hybridization. In the genus, diploidy is rare and correlated with sexual reproduction, whereas polyploidy and apomixis prevail (Valle and Savidan, 1996), which makes cross breeding difficult. In addition, apomixis and polyploidy represent challenges to genomic studies, particularly polyploidy, due to the increased complexity of the genome. In the following sections, we summarized the knowledge regarding these two peculiar characteristics of *Urochloa* spp.

### Polyploidy

A polyploid individual has a genome with more than two homologous sets of chromosomes, which is a condition that is quite common in all groups of plants and one of the major forces in plant evolution and speciation (Hegarty et al., 2013; Mason and Wendel, 2020). Polyploids often have larger cells in relation to their ancestral diploids, which can cause an increase in the whole size of the plant (“giga effect”; Stebbins, 1950), in addition to other advantages in genome buffering, vigorousness, and robust adaptation to environmental changes (Tamayo-Ordóñez et al., 2016). From a genetic point of view, polyploids have multiple alleles associated with a single locus, which implies high heterozygosity and more complex segregation compared with that observed with diploids (Schaart et al., 2021), and thus, the traditional breeding of polyploid crops is markedly more challenging.

Many important forage grasses are natural polyploid species, and others have chromosomes artificially duplicated by the use of colchicine, a chemical agent that can be used to produce artificial polyploids on demand. The induction of polyploidy can be an important tool in the genetic improvement programs of some species, which have three basic purposes: polyploidization of a hybrid to restore its fertility or synthesize a new species; polyploidization to obtain a larger and better plant (“giga effect”); and polyploidization to overcome the barrier imposed by different ploidy levels and allow intra- or interspecific crossing (Dewey, 1980; Sattler et al., 2016).

In the *U. ruziziensis*/*U. decumbens*/*U. brizantha* agamic complex, even though sexual individuals are known only in the wild as diploids, they have artificially duplicated chromosomes, which results in the induction of autotetraploids (Gobbe et al., 1981; Swenne et al., 1981; Pinheiro et al., 2000; Simioni and Valle, 2009; Timbó et al., 2014b). These polyploidized sexual individuals are essential for hybridization with apomictic individuals in breeding programs, which allows intra- and interspecific crossings and result in increased genetic variability and enhanced selection.

Natural polyploid species may originate from genomic duplication within a single species, which leads to autopolyploids, or by a hybridization of two closely related species (“subgenomes”), which results in allopolyploids. The identification of each class is frequently accomplished by analyzing meiotic pairing and inheritance patterns: autopolyploids exhibit polysomic inheritance, with pairing and recombination between all homologous copies of each chromosome, whereas allopolyploids display disomic inheritance, in which more-related chromosomes (“homologs”) may pair and recombine more frequently than less-related chromosomes (“homeologs”). However, in many cases, the evolutionary history is more complex, and pairing between related chromosomes from different subgenomes (“homeologous”) occurs to a limited extent. In such cases, individuals are classified as segmental allopolyploids (Bourke et al., 2018a; Mason and Wendel, 2020; Schaart et al., 2021).

Meiotic studies and molecular karyotyping of cultivar and hybrid genera have revealed several abnormalities typical of autopolyploid and segmental allopolyploid in U. decumbens (Mendes-Bonato et al., 2001, 2002b; Worthington et al., 2016), *U. brizantha* (Mendes-Bonato et al., 2002a, 2006; Mendes et al., 2006), *U. ruziziensis* (Mendes-Bonato et al., 2006), and *U. humidicola* (Vigna et al., 2016a; Worthington et al., 2019). These abnormalities, such as distorted chromosome segregation, asynchrony during microsporogenesis, preferential (intragenomic) pairing, and the presence of multivalent and micronuclei, demonstrate the segmental allopolyploid composition of these genomes.

Based on genomic *in situ* hybridization, de Paula et al. (2017) recently suggested the allopolyploid origin of *U. brizantha* and *U. decumbens*, and such results were validated by additional GISH analysis (Corrêa et al., 2020). The authors proposed genomic compositions of BBB^1^B^1^ for *U. brizantha*, B^1^B^1^B^2^B^2^ for *U. decumbens*, and B^2^B^2^ for the diploid *U. ruziziensis*. The findings indicate *U. ruziziensis* as the ancestral donor of genome B^2^, while the origin of genomes B and B^1^ remains unknown, but eligible candidates would be diploid genotypes of *U. brizantha* and *U. decumbens*. The probes used in GISH experiments reveal the homology between the three genomes (B, B^1^ and B^2^) and highlight the greater proximity between all three species, as previously suggested (Pessoa-Filho et al., 2017; Triviño et al., 2017).

An allopolyploid is not strictly the sum of two different genotypes, and most studies that investigated the process of polyploidization observed that genomes usually undergo a series of evolutionary processes after polyploidization, such as natural chromosome duplication, genome reorganization, changes in the gene expression pattern, and sub- and/or neofunctionalization of duplicate genes (Chen and Ni, 2006; Renny-Byfield and Wendel, 2014). A study performed using meiotic analyses and SSR amplification patterns exemplified the occurrence of such processes in *U. humidicola* (Vigna et al., 2016a). The authors suggested the allopolyploid origin of the genotypes H031 and cv. BRS Tupi from a cross between two different genomes, resulting in a triploid parent (2n = 3x = 18, ABB) that, after natural chromosome duplication, formed an allohexaploid (2n = 6x = 36, AABBBB).

Worthington et al. (2019) reinforced the thesis of AABBBB constitution for the apomictic genotype CIAT 16888; however, linkage and molecular karyotyping results showed no evidence of subgenome differentiation in the H031 genotype (CIAT 26146). More studies on the genomic composition of the sexual genotype H031 are needed. As noted by Worthington et al. (2019), if the sexual and other apomictic accessions of *U. humidicola* do indeed have different genomic compositions, H031 may need to be classified as a separate species or subspecies.

Despite the widespread occurrence of polyploidy in the *Urochloa* genus, many questions remain unanswered, which demonstrates the need to conduct additional research to understand the genome composition and evolution in tropical forage grasses. A high-quality reference genome is also fundamental to capture the variation and to better understand these economically important genomes (Tang, 2017; Kyriakidou et al., 2018). The last decades have seen remarkable advances in the field of polyploidy, particularly in molecular breeding approaches that combine QTL, GWAS, and GS models with allele dosage information (Sattler et al., 2016) and in tools for improving the genome sequencing and assembly of polyploid plant crops (Jiao and Schneeberger, 2017). We hope that these tools can aid a deeper understanding of the complexities of genetic variation that underlie the phenotype, which would allow plant breeders to precisely manipulate the genomes of *Urochloa* species to achieve outstanding results.

### Apomixis

Apomixis is defined as an asexual reproductive mode in which the formation of seeds occurs without fertilization, which results in exact genetic replicas (clones) of the female parent (Asker and Jerling, 1992; Hand and Koltunow, 2014). This condition allows us to capture, fix, and propagate the hybrid vigor of superior heterozygous genotypes per seed (Sailer et al., 2016) in a simpler manner compared with vegetative propagation or the production of hybrid seeds from inbred parental lines (Worthington et al., 2016). Several studies have focused on investigating the genetic control of this reproductive mode for practical applications, particularly in agriculture, but much knowledge remains unknown (Hand and Koltunow, 2014; Kaushal et al., 2019; Schmidt, 2020).

In the *Urochloa* genus, the type of apomixis is aposporic gametophytic, in which fertilization of the endosperm (pseudogamy) is necessary (do Valle, 1990; Lutts et al., 1994). Traditionally, the characterization of the reproductive mode of *Urochloa* species is conducted by embryo sac analysis, which is a very expensive and laborious method that requires adult plants for evaluation. Sexual and apomictic embryo sacs are often found on the same plant or even in the same ovule, and the observation of at least one aposporic embryonic sac indicates facultative apomixis (do Valle, 1990).

Most studies that investigated the inheritance of apomixis concluded that it is simply inherited and that the trait is conferred by a single dominant Mendelian factor (Valle et al., 1994; Miles and Escandón, 1997; Pessino et al., 1997, 2004), denoted as the ASGR (Ozias-Akins and van Dijk, 2007). Therefore, in crossbreeding, progeny segregates to the reproductive mode on a 1:1 basis (Valle and Savidan, 1996; Barrios et al., 2013; Worthington and Miles, 2015; Vigna et al., 2016a).

Studies were performed to investigate the chromosomal duplication of sexual accessions in some *Urochloa* species (Swenne et al., 1981; Pinheiro et al., 2000; Simioni and Valle, 2009). In addition, the genetic diversity maintained in apomictic accessions has recently started to be explored in breeding programs through crosses between apomictic (used as pollen donors) and sexual genotypes (Miles, 2007). In addition to increasing genetic variability, these crosses allow the selection of superior genotypes (Simeão et al., 2021). The progeny obtained from these crosses need to be phenotyped with respect to the reproductive mode with the aim of the next hybridizations. Sexual offspring can be used as female parents in future crosses, and apomictic offspring are potential cultivars as well as possible male parents. Therefore, the search for markers linked to apomixis is one of the main demands of breeding programs for the MAS of hybrids, mainly in Brazilian programs. The use of molecular markers that cosegregate with apomixis represents enormous potential and enables the rapid screening of thousands of progenies at the seedling stage (Worthington and Miles, 2015).

In the CIAT breeding program, MAS for apomixis has been performed since 2009 based on a SCAR marker called “N14.” This marker was developed from a RAPD marker linked to apomixis in *U. decumbens* cv. Basilisk and *U. brizantha* cv. La Libertad (Pedraza Garcia, 1995) and is able to detect apomixis mainly when cv. Basilisk is used as the pollen donor parent. For more genetically distant species, such as *U. humidicola*, this marker is not effective (Worthington et al., 2016).

Comparative molecular analyses and cytological studies have revealed that the region that controls apomixis (ACR) is apparently located in a chromosomal region with no recombination events documented to date (Ortiz et al., 2013). Therefore, this genomic structure exhibits a high level of conservation among apomictic species. For instance, the synteny of this region has already been found for *Urochloa* with rice and corn (Ozias-Akins and van Dijk, 2007).

To date, genetic mapping is one of the most frequent strategies used in investigating apomixis at the molecular level and in the search for markers intrinsically linked to this characteristic in *Urochloa* spp. Pessino et al. (1997, 1998) genotyped *Urochloa* hybrids with AFLP and RFLP markers identified in maize and rice and observed a total of nine markers linked to apomixis in *U. brizantha* cv. Marandu and the close synteny of some markers to the region of maize chromosome 5 and rice chromosome 2. A RAPD marker was identified as linked to apomixis in *U. humidicola* (Zorzatto et al., 2010). Additionally, in *U. humidicola*, Vigna et al. (2016a) mapped the locus that controls apospory in LG2, which is located 19.4 cM from the nearest SSR marker, and this finding corroborates the results reported by Pessino et al. (1998).

The ASGR was mapped to *U. decumbens* chromosome 5 in a region showing synteny with foxtail millet chromosome 5. In addition, the *PsASGR-BABY BOOM-like* (*psASGR–BBML*)-specific primer (p779/p780), whose candidate gene for the apomixis parthenogenesis component was first described in *Pennisetum squamulatum* (Ozias-Akins et al., 1998), was linked with ASGR, which indicates that this region is highly conserved across Paniceae species (Worthington et al., 2016). Following this same strategy, Worthington et al. (2019) mapped the ASGR in *U. humidicola* chromosome 1, a region syntenic with chromosomes 1 and 7 of *foxtail millet*. Although the ASGR was mapped to a different carrier chromosome than previously identified in *U. decumbens*, this study reinforced the postulation of ASGR-BBML as candidate genes for the parthenogenesis component of apomixis.

Some studies have suggested that events related to apomixis occur due to the dysregulation or suppression of genes involved in sexual reproduction (Ozias-Akins and van Dijk, 2007) or to rearrangements in the expression of genes that constitute the sexual pathway (Grimanelli et al., 2001). Thus, genetic studies have improved the understanding of the molecular mechanism of the different modes of reproduction in *Urochloa* species, which have particularly focused on apomixis (Rodrigues et al., 2003; Alves et al., 2007; Silveira et al., 2009, 2012; Lacerda et al., 2012; Guimarães et al., 2013; Ferreira et al., 2017; Koehler et al., 2020). Most of these studies were performed with *U. brizantha*, and the most recent study concluded that somatic embryogenesis receptor-like kinase (SERK) genes present different expression during somatic embryogenesis and sporogenesis of sexual and apomictic genotypes, which suggests a possible downregulation associated with apomictic development (Koehler et al., 2020).

These studies at the gene level open new perspectives for the transfer of apomixis to sexual plants in the future *via* gene editing, but other genomic approaches, including bacterial artificial chromosomes (BACs), should be applied for the identification and characterization of the genomic regions that control apomixis in *Urochloa* spp. In addition, a greater understanding of the genetic basis and regulatory mechanisms underlying apomictic seed development, including epigenetic mechanisms, is needed. In addition, efforts to develop a diagnostic marker test for apomixis are still highly desirable for the early identification of apomixis because it can shorten selection cycles and reduce the costs of breeding programs.

## Conclusion and Prospects

At present, to maintain and increase the use of tropical forage grasses as pastures in livestock agriculture, there is a continued need for improvements in the biomass and seed yield, nutritional quality, disease and pest resistance, and tolerance to abiotic stresses, including drought, in a more sustainable manner. The breeding programs of *Urochloa* spp. are quite recent, and knowledge about the genetics and genomics of the main species remains in progress. Moreover, genomic studies must be guided according to the specific objectives in the improvement of each species. Thus far, the basic knowledge of the *Urochloa* spp. genomes has been obtained through cytological studies, molecular markers, linkage maps and transcriptome sequences, and this information was obtained more slowly compared with that of other crops due to low investments and genomic complexities, including polyploidy.

Further studies of *Urochloa* spp. are still needed to understand and mine the genomic composition and diversity in general and to elucidate the genetic responses to different abiotic and biotic stress conditions. In addition, there is a strong demand for diagnostic molecular markers for target traits, particularly apomixis and spittlebug resistance. Currently, the opportunities to enrich the genomic knowledge of *Urochloa* spp. and accelerate breeding progress are growing with the advancement of technologies and computational methods, including efforts to explore and dissect complex traits of agronomic relevance. GWASs and GS studies remain in their infancy and should consider large panels of SNP markers and high-throughput phenotyping. In addition, due to the biology of *Urochloa* spp., more suitable models for the prediction of hybrid performance are needed, and deep learning techniques show great promise for this purpose. Moreover, specific strategies that consider the species, reproductive system, genome structure, and breeding purposes will have to be developed for GS.

Diploid reference genomes of *Urochloa ruziziensis* were recently made available (Pessoa-Filho et al., 2019; Worthington et al., 2021), which represents an important advance in the genomic resources for successfully leveraging genomic and molecular studies and breeding strategies, including GS. However, it is necessary to explore this and future genome assemblies in combination with different “omics” tools, such as metabolomics, transcriptomics and phenomics, which would ensure a better understanding of the expression and architecture of complex traits in Urochloa species. Thus, it is necessary to increase the research efforts aiming to develop innovative strategies that can be used in both basic research and applied improvements to generate new superior cultivars more effectively and guarantee a positive future impact on livestock production. Also, to make available genomic resources even more accessible and useful, an user-friendly genome browser for *Urochloa* and other tropical forage grass species should be implemented, allowing researchers to visually compare and correlate information from several different genomic sources.

In this review, we focused on the main characteristics of the four main species of *Urochloa* used in pastures, and we emphasized the genomic resources and molecular tools available for these species. This review should be helpful for scientists applying biological, genetic, and genomic approaches in studies of tropical forage grass species.

## Author Contributions

RF, AC, and AS jointly conceived this review. RF and AC prepared the first draft. LC, BV, and RS critically revised the manuscript. AS supervised the project. All authors read and approved the final manuscript.

## Funding

This work was supported by grants from the Fundação de Amparo à Pesquisa de do Estado de São Paulo (FAPESP), Coordenação de Aperfeiçoamento de Pessoal de Nível Superior (CAPES) and the Conselho Nacional de Desenvolvimento Científico e Tecnológico (CNPq). RF received a PD fellowship from FAPESP (2018/19219–6), and AS received research fellowships from CNPq (312777/2018–3), CAPES - Computational Biology program (88882.160095/2013–01) and FAPESP (2008/52197-4, 2005/51010-0).

## Conflict of Interest

The authors declare that this review was written in the absence of any commercial or financial relationships that could be construed as a potential conflict of interest.

## Publisher’s Note

All claims expressed in this article are solely those of the authors and do not necessarily represent those of their affiliated organizations, or those of the publisher, the editors and the reviewers. Any product that may be evaluated in this article, or claim that may be made by its manufacturer, is not guaranteed or endorsed by the publisher.

## Footnotes

^1^ https://www.ncbi.nlm.nih.gov/assembly/?term=urochloa%20ruziziensis

^2^ http://www.fao.org/faostat/en/#data
